# Mortality Among Patients With Early-Onset Atrial Fibrillation and Rare Variants in Cardiomyopathy and Arrhythmia Genes

**DOI:** 10.1001/jamacardio.2022.0810

**Published:** 2022-05-11

**Authors:** Zachary T. Yoneda, Katherine C. Anderson, Fei Ye, Joseph A. Quintana, Matthew J. O’Neill, Richard A. Sims, Lili Sun, Andrew M. Glazer, Giovanni Davogustto, Majd El-Harasis, James L. Laws, Brittany N. Saldivar, Diane M. Crawford, Thomas Stricker, Quinn Wells, Dawood Darbar, Gregory F. Michaud, Lynne W. Stevenson, Steven A. Lubitz, Patrick T. Ellinor, Dan M. Roden, M. Benjamin Shoemaker

**Affiliations:** 1 Department of Medicine, Division of Cardiovascular Medicine, Vanderbilt University Medical Center, Nashville, Tennessee; 2 Department of Biostatistics, Vanderbilt University Medical Center, Nashville, Tennessee; 3 Vanderbilt University School of Medicine, Nashville, Tennessee; 4 Department of Medicine, Division of Clinical Pharmacology, Vanderbilt University Medical Center, Nashville, Tennessee; 5 Department of Pathology, Microbiology and Immunology, Vanderbilt University Medical Center, Nashville, Tennessee; 6 Department of Pharmacology, Vanderbilt University Medical Center, Nashville, Tennessee; 7 Department of Biomedical Informatics, Vanderbilt University Medical Center, Nashville, Tennessee; 8 Department of Medicine, Division of Cardiology, University of Illinois at Chicago; 9 Cardiovascular Disease Initiative, The Broad Institute of MIT and Harvard, Cambridge, Massachusetts; 10 Cardiovascular Research Center & Demoulas Center for Cardiac Arrhythmias, Massachusetts General Hospital, Boston

## Abstract

**Importance:**

Patients with early-onset atrial fibrillation (AF) are enriched for rare variants in cardiomyopathy and arrhythmia genes. The clinical significance of these rare variants in patients with early-onset AF is unknown.

**Objective:**

To assess the association between rare variants in cardiomyopathy and arrhythmia genes detected in patients with early-onset AF and time to death.

**Design, Setting, and Participants:**

This prospective cohort study included participants with AF diagnosed before 66 years of age who underwent whole-genome sequencing through the National Heart, Lung and Blood Institute’s Trans-Omics for Precision Medicine program. Participants were enrolled from November 23, 1999, to June 2, 2015. Data were analyzed from February 26 to September 19, 2021.

**Exposures:**

Rare variants identified in a panel of 145 genes that are included in cardiomyopathy and arrhythmia panels used by commercial clinical genetic testing laboratories.

**Main Outcomes and Measures:**

The primary study outcome was time to death and was adjudicated from medical records and the National Death Index. Multivariable Cox proportional hazards regression was used to evaluate the association of disease-associated variants with risk of death after adjustment for age at AF diagnosis, sex, race, body mass index, left ventricular ejection fraction, and an interaction term of age at AF diagnosis and disease-associated variant status.

**Results:**

Among 1293 participants (934 [72%] male; median age at enrollment, 56.0 years; IQR, 48.0-61.0 years), disease-associated (pathogenic or likely pathogenic) rare variants were found in 131 (10%). During a median follow-up of 9.9 years (IQR, 6.9-13.2 years), 219 participants (17%) died. In univariable analysis, disease-associated variants were associated with an increased risk of mortality (hazard ratio, [HR], 1.5; 95% CI, 1.0-2.1; *P* = .05); the association remained significant in multivariable modeling when adjusted for age at AF diagnosis, sex, race, body mass index, left ventricular ejection fraction, and an interaction term between disease-associated variant status and age at AF diagnosis. The interaction demonstrated that disease-associated variants were associated with a significantly higher risk of mortality compared with no disease-associated variant when AF was diagnosed at a younger age (*P* = .008 for interaction). Higher body mass index (per IQR: HR, 1.4; 95% CI, 1.2-1.6; *P* < .001) and lower left ventricular ejection fraction (per IQR: HR, 0.8; 95% CI, 0.7-0.8; *P* < .001) were associated with higher mortality risk. There were 73 cardiomyopathy-related deaths, 40 sudden deaths, and 10 stroke-related deaths. Mortality among patients with the most prevalent genes with disease-associated variants was 26% (10 of 38 patients) for *TTN*, 33% (6 of 18) for *MYH7*, 22% (2 of 9) for *LMNA*, 0% (0 of 10) for *MYH6*, and 0% (0 of 8) for *KCNQ1*.

**Conclusions and Relevance:**

The findings suggest that rare variants in cardiomyopathy and arrhythmia genes may be associated with increased risk of mortality among patients with early-onset AF, especially those diagnosed at a younger age. Genetic testing may provide important prognostic information for patients with early-onset AF.

## Key Points

**Question:**

In patients with early-onset atrial fibrillation (AF), are rare variants in cardiomyopathy and arrhythmia genes associated with increased all-cause mortality?

**Findings:**

In this cohort study of 1293 participants diagnosed with AF before 66 years of age, time to death was significantly associated with a disease-associated variant, age at AF diagnosis, and the interaction between age at AF diagnosis and variant status.

**Meaning:**

The findings suggest that among patients with early-onset AF, the presence of a disease-associated rare variant for an inherited cardiomyopathy or arrhythmia syndrome may be associated with an increased risk of mortality.

## Teaser

This cohort study assesses the association of rare variants of cardiomyopathy and arrhythmia genes with all-cause mortality among patients with early-onset atrial fibrillation.

## Introduction

The independent association between atrial fibrillation (AF) and increased risk of mortality is well established in older populations.^1,2,3,4^ Less well-studied are younger patients who are diagnosed with AF despite clinical risk factors. Patients with idiopathic or lone AF were first reported^5^ to have a higher risk of mortality almost 40 years ago, but the pathophysiology was unknown.^6^ The recognition that patients with early-onset AF are enriched for rare genetic variants associated with inherited cardiomyopathy (CM) and arrhythmia syndromes suggests that in some patients, AF may be the first sign of a more serious underlying genetic disease.^7,8,9,10^ A study^11^ recently reported that sequencing of a 145-gene CM and arrhythmia panel identified pathogenic or likely pathogenic variants in 10.1% of patients diagnosed with AF before 66 years of age and 16.8% of those diagnosed before 30 years of age.

The effect of rare genetic variants on clinical outcomes and long-term prognosis in patients with early-onset AF remains undefined. Among patients with hypertrophic CM (HCM), dilated CM (DCM), Brugada syndrome, and long QT syndrome, genotype-positive patients have been consistently shown to have worse clinical outcomes, such as a higher risk for malignant ventricular arrhythmias, progression to end-stage heart failure, and mortality.^12,13,14,15,16,17,18,19^ Accordingly, we hypothesized that disease-associated rare variants in patients with early-onset AF would be associated with a higher risk of mortality and that there would be an interaction between genotype-positive status and younger age at AF diagnosis in terms of mortality risk. We assessed a cohort of 1293 patients with early-onset AF who underwent whole-genome sequencing^11^ and searched the Centers for Disease Control and Prevention National Death Index (NDI) to identify participants who died during follow-up.

## Methods

### Study Population

This cohort study included participants enrolled in the Vanderbilt Atrial Fibrillation Registry or Vanderbilt AF Ablation Registry from November 23, 1999, to June 2, 2015 (eAppendix 1 in the Supplement). Eligible participants were diagnosed with AF before 66 years of age and underwent whole-genome sequencing through the National Heart, Lung and Blood Institute’s Trans-Omics for Precision Medicine Program as previously described.^7,11^ All participants provided written informed consent, and ethical approval for the study was obtained from the Vanderbilt University Medical Center institutional review board. Baseline demographic and clinical characteristics were recorded at the time of enrollment (eAppendix 2 in the Supplement). This study followed the Strengthening the Reporting of Observational Studies in Epidemiology (STROBE) reporting guideline.

### Whole-Genome Sequencing and Annotation

The sequencing methods and quality control steps for the Trans-Omics for Precision Medicine Program AF Project have been previously described.^7^ The methods for annotation and variant classification for this analysis have also been previously described.^11^ In brief, 145 CM and arrhythmia genes that were included in commercial genetic testing panels were selected (eTable 1 in the Supplement). An automated artificial intelligence-based process (Franklin by Genoox^20^) and subsequent manual adjudication were used to classify variants according to American College of Medical Genetics and Genomics (ACMG) criteria as benign, likely benign, variant of undetermined significant, likely pathogenic, or pathogenic.^21^ Participants with a pathogenic or likely pathogenic variant for an autosomal dominant or X-linked dominant syndrome were defined as having a disease-associated rare variant.

### Mortality and Cause of Death Assessment

A complete description of the methods for mortality and cause of death assessment are given in eAppendix 3 in the Supplement. In brief, a combination of manual review of the participants’ records and query of the NDI was performed to determine vital status, which was censored up to January 1, 2020. The NDI is the most complete source of death information in the US and includes all death certificates since 1979, with *International Statistical Classification of Diseases and Related Health Problems, Tenth Revision (ICD-10)* codes listed for causes of death. Deaths were considered CM-related, sudden deaths, or stroke-related based on inclusion of a corresponding *ICD-10* code (eTable 2 in the Supplement) as the primary or any of the contributing causes of death.

### Statistical Analysis

Data were analyzed from February 26 to September 19, 2021. A complete description of the statistical methods is given in eAppendix 4 in the Supplement. In brief, the primary outcome was time to from AF diagnosis to death or censor date, measured in days. The primary exposure was the presence or absence of a disease-associated variant. A univariable analysis used a Cox proportional hazards regression model to test the association between disease-associated variant status and the primary outcome. The primary analysis used a multivariable Cox proportional hazards regression model and tested the hypothesis that a disease-associated variant would be associated with increased risk of death after adjustment for age at AF diagnosis, sex, race, body mas index (BMI; calculated as weight in kilograms divided by height in meters squared), left ventricular ejection fraction (LVEF), and an interaction term of age at AF diagnosis and disease-associated variant status. A significant interaction term indicated that the association of disease-associated variants with risk of mortality differed for patients diagnosed at different ages. Partial-effects plots were then created to describe the interaction effect on risk of mortality, and the interaction *P* value is reported. Prespecified secondary analyses grouped disease-associated variants according to genes associated with specific inherited CM and arrhythmia syndromes and individual genes. A second prespecified subgroup analysis tested the association between disease-associated variants and specific causes of death (CM-related or sudden death). A sensitivity analysis tested whether presence of disease-associated variants restricted to a smaller gene panel composed of only strong or definitive evidence genes (eTable 3 in the Supplement) was associated with all-cause mortality using the same multivariable Cox proportional hazards regression model as in the primary analysis.

## Results

### Description of the Study Population

**Figure 1.**
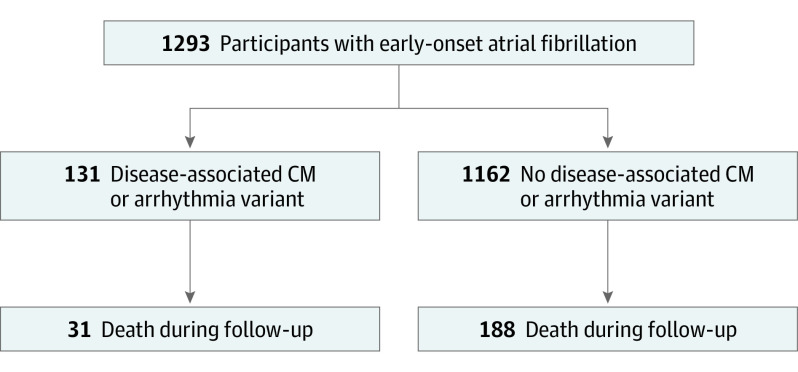
Breakdown of Participants According to Genotype Status and Death During Follow-up CM indicates cardiomyopathy.

There were 1293 participants (934 [72%] male; median age at enrollment, 56.0 years; IQR, 48.0-61.0 years) with early-onset AF enrolled from 1999 to 2015 who underwent whole-genome sequencing. Of those, 219 died during follow-up (16.9%) (Figure 1). The median duration of follow-up was 9.9 years (IQR, 6.9-13.2 years) from the time of enrollment and 15.0 years (IQR, 10.3-20.0) from the time of AF diagnosis. Demographics and clinical characteristics at enrollment are stratified by vital status in the Table.

**Table.** Demographics, Genetics, and Clinical Characteristics of Patients According to Mortality

Characteristic	Patients^a^		
	Total (N = 1293)	Deceased (n = 219)	Alive (n = 1074)
Age at enrollment, y			
Median (IQR)	56.0 (48.0-61.0)	59.0 (54.0-64.0)	54.0 (47.0-60.0)
<30	52 (4)	0	52 (5)
30-39	96 (7)	7 (3)	89 (8)
40-49	225 (17)	23 (11)	202 (19)
50-59	521 (40)	83 (38)	438 (41)
60-64	267 (21)	64 (29)	203 (19)
≥65	132 (10)	42 (19)	90 (8)
Age at AF diagnosis, y			
Median (IQR)	50.0 (41.0-56.0)	54.0 (47.5-57.0)	49.0 (40.0-55.0)
<30	119 (9)	10 (5)	109 (10)
30-39	146 (11)	17 (8)	129 (12)
40-49	366 (28)	40 (18)	326 (30)
50-59	553 (43)	133 (61)	420 (39)
60-65	109 (8)	19 (9)	90 (8)
Sex			
Female	359 (28)	57 (26)	302 (28)
Male	934 (72)	162 (74)	772 (72)
Self-reported race			
White	1238 (96)	208 (95)	1030 (96)
Black	48 (4)	11 (5)	37 (3)
Other^b^	7 (1)	0	7 (1)
Self-reported ethnicity			
Not Hispanic/Latinx	1286 (99.5)	218 (100)	1068 (99)
Hispanic/Latinx	7 (1)	1 (0)	6 (1)
Height, cm	177.8 (170.2-185.4)	177.8 (169.9-182.9)	178.0 (170.2-185.4)
Body mass index^c^			
Median (IQR)	30.3 (26.6-35.3)	31.4 (26.6-37.7)	30.1 (26.6-34.6)
<30	602 (48)	88 (42)	514 (50)
30-39	512 (41)	83 (39)	429 (41)
≥40	134 (11)	41 (19)	93 (9)
Hypertension	750 (58)	166 (76)	584 (54)
Valve disease	77 (6.0)	39 (17.8)	38 (3.5)
Myocardial infarction	118 (9.1)	58 (26.5)	60 (5.6)
Left ventricular ejection fraction, %			
Median (IQR)	55.0 (53.0-60.0)	55.0 (42.9-55.0)	55 (55.0-60.0)
<40	107 (9)	45 (21)	62 (6)
40-49	82 (7)	23 (11)	59 (6)
≥50	1062 (85)	144 (68)	918 (88)
Disease-associated rare variant	131 (10)	31 (14)	100 (9)
VUS, No.			
0	254 (20)	49 (22)	205 (19)
1	240 (19)	28 (13)	212 (20)
2	265 (20)	48 (22)	217 (20)
3	230 (18)	46 (21)	184 (17)
4	161 (12)	24 (11)	137 (13)
>4	143 (11)	24 (11)	119 (11)
Duration of follow-up or survival, median (IQR), y	9.9 (6.9-13.2)	6.5 (3.0-10.4)	10.6 (7.7-13.8)

Abbreviations: AF, atrial fibrillation; VUS, variant of undetermined significance.

^a^ Data are presented as number (percentage) of patients unless otherwise indicated.

^b^ Other included American Indian/Alaska Native and Asian/Pacific Islander individuals.

^c^ Calculated as weight in kilograms divided by height in meters squared.

### Results of ACMG Variant Classification

Full results of the ACMG variant classification have been previously published.^11^ Overall, a disease-associated rare variant was detected in 131 participants (10%). Of these, 93 participants carried a disease-associated variant in a DCM gene, 43 in an HCM gene, and 37 in an arrhythmogenic CM or arrhythmogenic right ventricular CM gene. There was considerable overlap between genes associated with DCM, HCM, and arrhythmogenic CM or arrhythmogenic right ventricular CM genes as shown in eTable 3 in the Supplement. For the channelopathies, 2 participants carried a disease-associated variant in a Brugada syndrome gene, 11 in a long QT syndrome gene, and 1 in a catecholaminergic polymorphic ventricular tachycardia gene. Overall, 8-times more participants carried CM variants (96 participants) than channelopathy variants (12 participants). Eight participants had 2 pathogenic or likely pathogenic variants, and 1 participant had 3 pathogenic or likely pathogenic variants.

### Association of Rare Variants With Mortality

**Figure 2.**
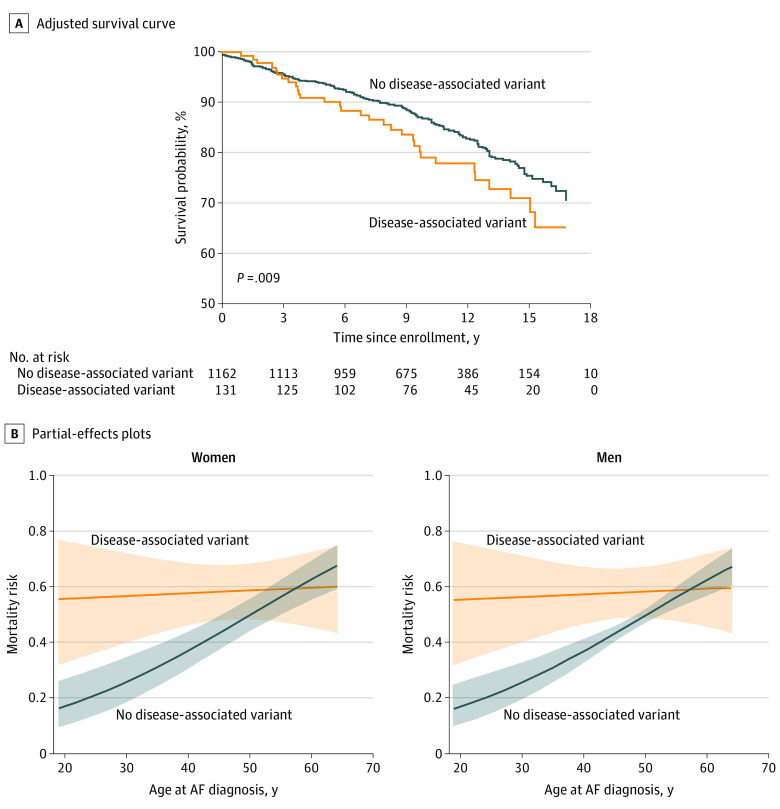
Adjusted Survival Curve of All-Cause Mortality Among Patients With Early-Onset Atrial Fibrillation (AF) With and Without Disease-Associated Rare Variants in Cardiomyopathy and Arrhythmia Genes and Interaction Between Age at AF Diagnosis and Disease-Associated Variants Shading indicates 95% CIs.

Among participants with a disease-associated variant, 31 (24%) died during follow-up compared with 188 participants (16%) without a disease-associated variant (Figure 1). In univariable analysis, presence of disease-associated variants was associated with an increased risk of all-cause mortality (HR, 1.5; 95% CI, 1.0-2.1; *P* = .05). In multivariable analysis, the presence of a disease-associated variant was associated with a significantly higher risk of all-cause mortality, as shown in the adjusted survival curve (χ^2^ = 9.39; *P* = .009 likelihood ratio test) (Figure 2A). The primary multivariable analysis demonstrated a statistically significant interaction between disease-associated variant status and age at AF diagnosis (*P =* .008 for interaction). Owing to this interaction, the estimates and 95% CIs for disease-associated variants are not presented separately, but instead using partial effects plots (Figure 2B). In the multivariable model, male sex (HR, 1.0; 95% CI, 0.7-1.4; *P* = .98), White race (HR, 1.1; 95% CI, 0.6-2.1; *P* = .74), increased BMI (HR, 1.4; IQR, 1.2-1.6; *P* = .001), and lower LVEF (HR, 0.8; 95% CI, 0.7-0.8; *P* < .001) were associated with increased mortality risk. In the analysis of the association of age with mortality, older age was associated with a higher risk of death for both genotype-positive and genotype-negative male and female participants. However, as shown in Figure 2B, the relative risk of death (distance between the 2 curves) was significantly higher for genotype-positive participants compared with genotype-negative participants among those diagnosed with AF at a younger age. This association diminished as age at diagnosis approached 60 years, where the curves crossed, and the relative risk of death became higher for genotype-negative participants.

### Association With Disease-Associated Variants Restricted to Strong or Definitive Evidence Genes

Results from the sensitivity analysis that restricted disease-associated variants to only those from strong or definitive evidence genes also demonstrated significant associations with mortality for disease-associated variants, age at AF diagnosis, BMI, and the interaction between disease-associated variants and age at AF diagnosis. Findings for race and ethnicity and sex remained nonsignificant. eFigure 1 in the Supplement shows the adjusted survival curve for this analysis.

### Association of Rare Variants With Causes of Death

**Figure 3.**
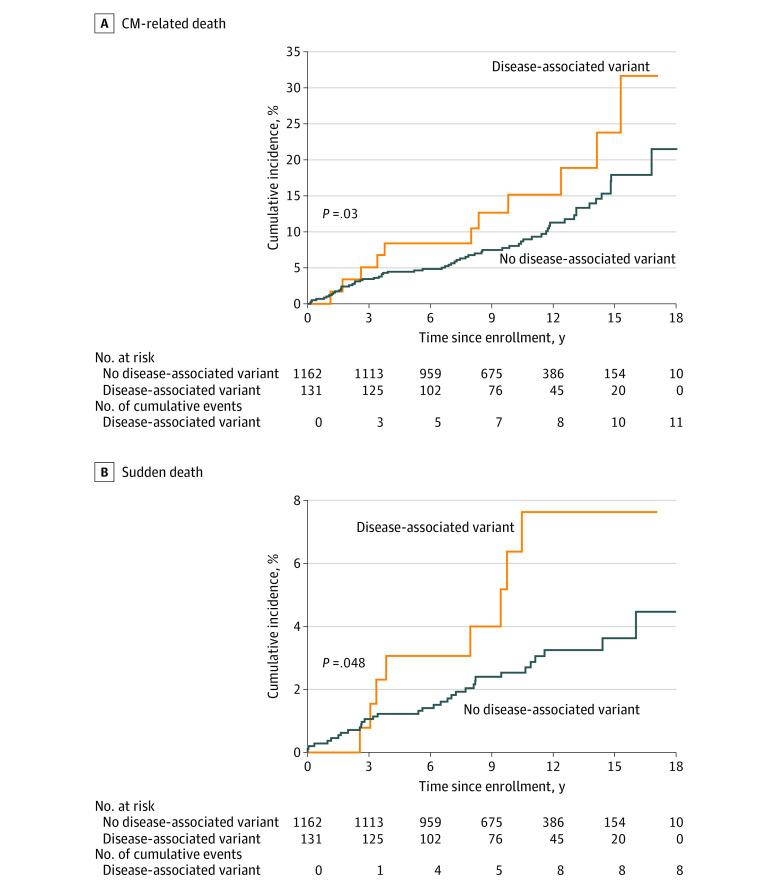
Adjusted Cumulative Incidence of Cardiomyopathy (CM)–Related Death and Adjusted Cumulative Incidence of Sudden Death According to Disease-Associated Rare Variant Status A, Adjustment was made for age at atrial fibrillation diagnosis, sex, race, body mass index, and the interaction between disease-associated variant status and age at atrial fibrillation diagnosis (*P* = .009 likelihood ratio test). B, Adjustment was made for age at atrial fibrillation diagnosis.

Of 219 deaths, 73 were CM-related (33%), 40 were sudden deaths (18%), and 10 were stroke-related (5%). The adjusted cumulative incidence plots of CM-related death and sudden death by variant status are presented in Figure 3. A multivariable Cox proportional hazards regression model was used to analyze CM-related deaths and sudden deaths. The risk of CM-related death was associated with disease-associated variant status, age at AF diagnosis, and the interaction between disease-associated variant status and age at AF diagnosis (*P* = .03 for interaction). This model was also adjusted for BMI (HR, 1.2; 95% CI, 1.0-1.5; *P* = .07), male sex (HR, 1.1; 95% CI, 0.7-1.6; *P* = .66), and White race (HR, 0.7; 95% CI, 0.3-1.4; *P* = .28). For sudden deaths, disease-associated variants (HR, 2.4; 95% CI, 1.1-5.2; *P* = .03) and age at AF diagnosis (HR, 2.4; 95% CI, 1.4-4.2; *P* = .002) were associated with a increased risk of mortality. The analysis of sudden death was not powered to test for an interaction. The data set was not powered to determine an association of variant status with time to stroke-related death.

**Figure 4.**
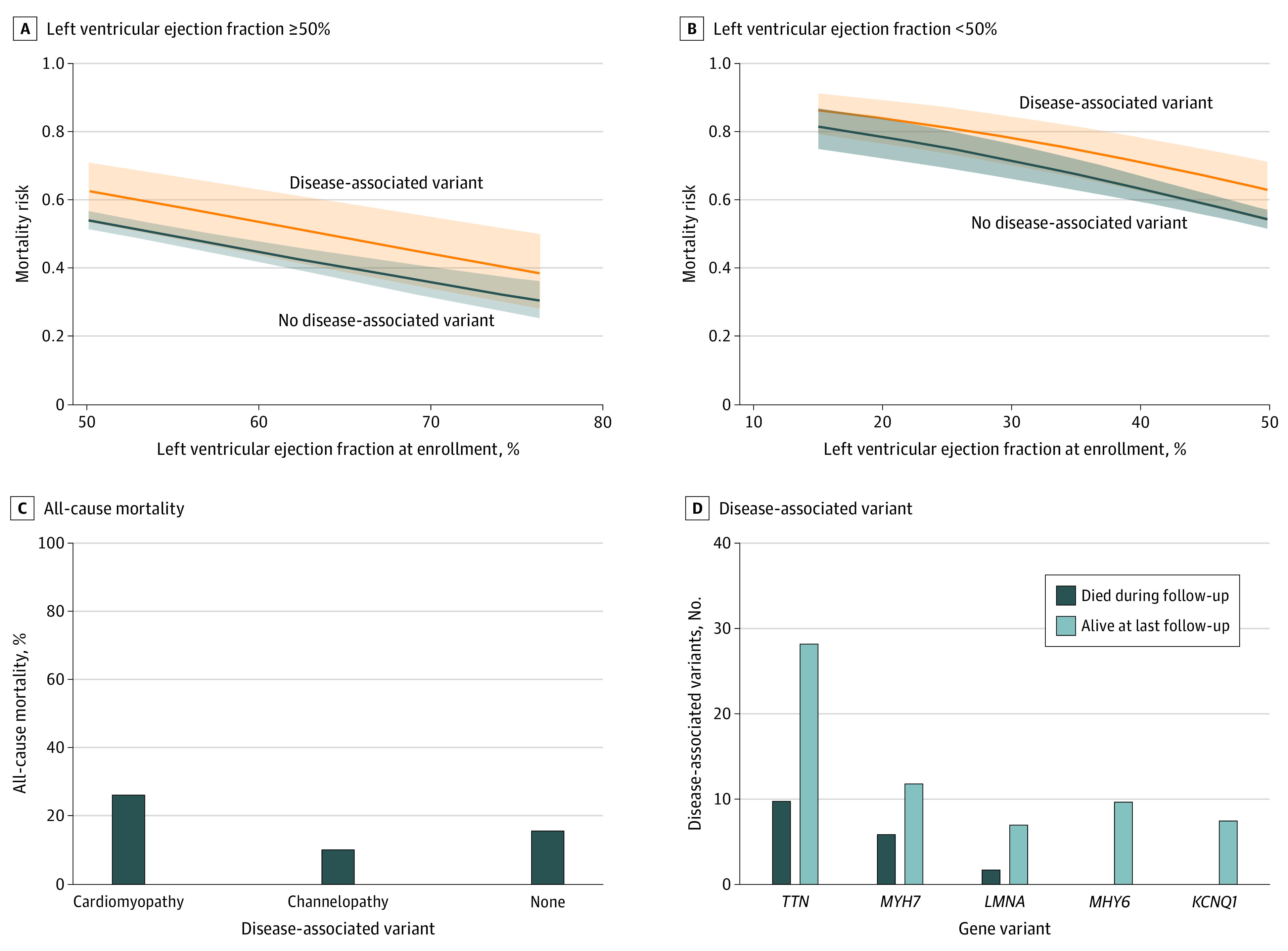
Disease-Associated Variants and Mortality Risk Among Participants A and B, Shading indicates 95% CIs.

Genetic variants were seen in all groups defined by baseline ejection fraction at enrollment. LVEF was associated with risk of mortality among participants with reduced LVEF (<50%) and preserved LVEF (≥50%) (Figure 4A and B). Most participants with a history of depressed left ventricular function at or before enrollment did not possess a disease-associated variant. The proportion with a disease-associated variant was 14% (15 of 107) in the group with LVEF of 39% or less, 12% (10 of 82) in the group with LVEF of 40% to 49%, and 9% (100 of 1062) in the group with LVEF of 50% of greater (eFigure 2 in the Supplement). Among participants with a disease-associated variant, the all-cause mortality was 47% (7 of 15) in the group with LVEF of 39% or less, 50% (5 of 10) in the group with LVEF of 40% to 49%, and 18% (18 of 100) in the group with LVEF of 50% or greater.

### All-Cause Mortality According to Genes Associated With CM and Channelopathy Syndromes and Individual Genes

Among the 92 participants with a disease-associated variant in any CM-related gene (DCM, HCM, or arrhythmogenic CM or arrhythmogenic right ventricular CM), there were 24 deaths (26%) compared with 1 death (10%) among the 10 participants with a disease-associated variant in a channelopathy-related gene (long QT syndrome, Brugada syndrome, or catecholaminergic polymorphic ventricular tachycardia) (Figure 4C). The deceased participant with a channelopathy variant had a heterozygous *CASQ2* variant. There were 2 participants with both a disease-associated CM variant and a channelopathy variant.

The most prevalent genes with disease-associated variants were *TTN* (OMIM 188840), *MYH7* (OMIM 160760), *MYH6* (OMIM 160710), *LMNA* (OMIM 150330), and *KCNQ1* (OMIM 607542). The number of deaths observed was 10 of 38 (26%) for *TTN*, 6 of 18 (33%) for *MYH7*, and 2 of 9 (22%) for *LMNA* compared with 0 of 8 for *KCNQ1* and 0 of 10 for *MYH6* (Figure 4D and eFigures 3-7 in the Supplement).

## Discussion

The association between AF and higher risk of mortality in younger patients with minimal clinical risk factors was previously unexplained.^5,6^ Our results demonstrated that among patients with early-onset AF, presence of disease-associated rare variants in CM and arrhythmia genes was associated with an approximately 1.5-fold higher risk of mortality over 10 years. Furthermore, there was a significant interaction such that the relative risk of mortality was greater when AF was diagnosed at a younger age among patients with a disease-associated rare variant. Among participants who died, the cause of death was more often CM-related (33%) than sudden death (18%) or stroke-related (5%), and the risks of CM-related and sudden death were significantly higher among patients with disease-associated variants. Most disease-associated variants in patients with early-onset AF were in CM genes. However, most of the participants who had reduced LVEF at the time of enrollment did not have a rare disease-associated variant. These data suggest that genetic testing may identify patients with a subtype of early-onset AF who are at higher risk of mortality, relative risk associated with a disease-associated variant may be higher when AF is diagnosed at a younger age, and relative risk may be associated predominantly with variants in CM-related genes.

### Age at AF Diagnosis and Risk of Mortality

Our results showed that age at AF diagnosis was significantly associated with a higher risk of mortality for both genotype-positive and genotype-negative participants. However, in genotype-positive participants, younger age at AF diagnosis was associated with significantly higher relative risk of mortality. This finding is consistent with the observation found across a variety of disorders that more deleterious genetic variation occurs in patients with younger age at disease onset. However, our data also demonstrated that as age at AF diagnosis approached 60 years, the mortality curves crossed such that genotype-negative participants started to have a higher risk of mortality. We suspect this finding was attributable to the clinical risk factors for AF found in the genotype-negative group (eg, high BMI and its related comorbidities) that were associated with higher risk of mortality at older age at AF diagnosis. However, the genetic variants associated with older age at AF diagnosis in the genotype-positive group may have been those that are less deleterious and have reduced penetrance.

### Mortality According to Specific Inherited CM and Arrhythmia Syndromes and Individual Genes

The gene panel used in this study was large and included genes associated with a variety of different syndromes with variable risks of mortality due to heart failure, sudden death, and stroke. To describe the effects of individual genes, we presented the mortality rate among patient with the most prevalent genes with disease-associated variants. Among the 38 participants with a disease-associated *TTN* variant, 10 (26%) died during follow-up. *TTN* encodes the sarcomeric protein titin and is the most prevalent genetic contributor to both early-onset AF^7,8^ and DCM.^22,23,24^ Among the 18 participants with a disease-associated *MYH7* variant, 6 (33%) died during follow-up. *MYH7* encodes the beta myosin heavy chain subunit and is expressed in the atria and ventricles. Disease-associated variants in *MYH7* are among the most prevalent genetic contributors to HCM^25^ and are associated with higher risk for AF than variants in *MYBPC3* and thin filament genes.^26,27^ Among the 9 participants with a disease-associated *LMNA* variant, 2 (22%) died during follow-up. *LMNA* encodes lamin A and C, which are structural proteins that comprise the inner nuclear membrane. Patients with *LMNA* variants can present with any combination of DCM, atrioventricular block, atrial or ventricular arrhythmias, or Emery-Dreifuss muscular dystrophy.^28,29,30,31^

In contrast to participants with variants in *TTN*, *MYH7,* and *LMNA*, none of the 10 participants with a disease-associated *MYH6* variant died during follow-up. *MYH6* encodes the alpha myosin heavy chain subunit and is predominantly expressed in the human atria.^32,33^ These results suggest that patients with early-onset AF associated with an *MYH6* variant may have more isolated atrial involvement compared with those with an *MYH7* variant and may have a genetic subtype of early-onset AF associated with lower risk of mortality. Similarly, none of the 8 participants with a disease-associated variant in *KCNQ1*, the gene associated with LQT type 1, died during follow-up. Larger studies are needed to validate and potentially explain the lower rate of mortality observed in *MYH6* and *KCNQ1* variant carriers compared with carriers of other genes.

### Deaths Related to Cardiomyopathy, Sudden Death, and Stroke

According to the primary or contributing causes of death as listed on death certificates, CM-related deaths (n = 73) were more common than sudden deaths (n = 40) and stroke-related deaths (n = 10). Furthermore, carrying a disease-associated rare variant was associated with a significantly higher risk of CM-related death and sudden death. Our results suggest that therapies to prevent sudden death and the progression of CM should be prioritized for further investigation to address the excess mortality observed among genotype-positive patients with early-onset AF.

### Limitations

This study has limitations. Although the primary analysis was sufficiently powered to demonstrate an association between disease-associated rare variants and mortality, subgroup analyses were underpowered and larger studies will be needed to define the risk according to variants for individual syndromes, individual genes, and in racial and ethnic minority groups.^9^ Specifically, most of the disease-associated variants in channelopathy genes were in *KCNQ1*; thus, it is unknown whether these results are generalizable to other channelopathy genes, such as *KCNH2*, *SCN5A*, and *RYR2*. Furthermore, although this study defined the association between rare variants and mortality among patients with early-onset AF by querying the NDI, future studies using deep phenotyping methods will be needed to better define clinical characteristics, such as CM subtypes. The reliability of cause of death information as listed on death certificates is a potential limitation, and thus the specific mechanism of death remains uncertain. Future studies will be needed to replicate these results, and prospective trials will be needed to investigate whether genetic testing in patients with early-onset AF improves clinical outcomes.

## Conclusions

In this study, patients with early-onset AF who carried a disease-associated CM or arrhythmia variant had a higher risk of all-cause mortality and a higher risk for CM-related death and sudden death. Compared with genotype-negative patients, those with a disease-associated variant had a higher relative risk of death when AF was diagnosed at a younger age. Most patients with a history of reduced LVEF at enrollment did not have a disease-associated variant, but among those who did, the mortality rate was 48% during a median 9.9 years of follow-up. Furthermore, the mortality rate among participants with preserved left ventricular function and a disease-associated variant was 18% during follow-up. These data suggest that there is prognostic value for genetic testing in patients with early-onset AF regardless of ejection fraction at the time of presentation. Future studies are needed to prospectively define whether genetic testing improves clinical outcomes in patients with early-onset AF.

## Notes

Supplement. **eAppendix 1.** Description of the Registries **eAppendix 2.** Definitions for Baseline Demographics and Clinical Characteristics **eTable 1.** Gene List Table **eAppendix 3.** Mortality and Cause of Death Assessment **eTable 2.** ICD-10 Codes Listed as Primary or Contributing Causes of Death From NDI Records **eAppendix 4.** Statistical Methods **eTable 3.** Genes for Which Disease-Associated Variants Were Detected Grouped According to Inherited CM and Arrhythmia Syndromes **eFigure 1.** Adjusted Survival Curve for the Sensitivity Analysis That Restricted Disease-Associated Variants to Only Strong/Definitive Evidence Genes **eFigure 2.** Proportion of Participants With a Disease-Associated Variant According to LVEF at Enrollment **eFigure 3.** Kaplan-Meier Curve for Survival According to Disease-Associated *TTN* Variants **eFigure 4.** Kaplan-Meier Curve for Survival According to Disease-Associated *MYH7* Variants **eFigure 5.** Kaplan-Meier Curve for Survival According to Disease-Associated *LMNA* Variants **eFigure 6.** Kaplan-Meier Curve for Survival According to Disease-Associated *MYH6* Variants **eFigure 7.** Kaplan-Meier Curve for Survival According to Disease-Associated *KCNQ1* Variants

## References

[1] Benjamin EJ, Wolf PA, D’Agostino RB, Silbershatz H, Kannel WB, Levy D. Impact of atrial fibrillation on the risk of death: the Framingham Heart Study. Circulation. 1998;98(10):946-952. doi:10.1161/01.CIR.98.10.946 9737513

[2] Krahn AD, Manfreda J, Tate RB, Mathewson FA, Cuddy TE. The natural history of atrial fibrillation: incidence, risk factors, and prognosis in the Manitoba Follow-Up Study. Am J Med. 1995;98(5):476-484. doi:10.1016/S0002-9343(99)80348-9 7733127

[3] Lake FR, Cullen KJ, de Klerk NH, McCall MG, Rosman DL. Atrial fibrillation and mortality in an elderly population. Aust N Z J Med. 1989;19(4):321-326. doi:10.1111/j.1445-5994.1989.tb00271.x 2789508

[4] Kannel WB, Abbott RD, Savage DD, McNamara PM. Epidemiologic features of chronic atrial fibrillation: the Framingham study. N Engl J Med. 1982;306(17):1018-1022. doi:10.1056/NEJM198204293061703 7062992

[5] Gajewski J, Singer RB. Mortality in an insured population with atrial fibrillation. JAMA. 1981;245(15):1540-1544. doi:10.1001/jama.1981.03310400022019 7206163

[6] Jouven X, Desnos M, Guerot C, Ducimetiere P. Idiopathic atrial fibrillation as a risk factor for mortality. The Paris Prospective Study I. Eur Heart J. 1999;20(12):896-899. doi:10.1053/euhj.1998.1397 10329095

[7] Choi SH, Weng LC, Roselli C, ; DiscovEHR study and the NHLBI Trans-Omics for Precision Medicine (TOPMed) Consortium. Association between titin loss-of-function variants and early-onset atrial fibrillation. JAMA. 2018;320(22):2354-2364. doi:10.1001/jama.2018.18179 30535219 PMC6436530

[8] Goodyer WR, Dunn K, Caleshu C, . Broad genetic testing in a clinical setting uncovers a high prevalence of titin loss-of-function variants in very early onset atrial fibrillation. Circ Genom Precis Med. 2019;12(11):e002713. doi:10.1161/CIRCGEN.119.002713 31638414 PMC10626994

[9] Chalazan B, Mol D, Darbar FA, . Association of rare genetic variants and early-onset atrial fibrillation in ethnic minority individuals. JAMA Cardiol. 2021;6(7):811-819. doi:10.1001/jamacardio.2021.0994 33950154 PMC8100900

[10] Yoneda ZT, Anderson KC, Estrada JC, . Genetic testing for early onset atrial arrhythmias changes clinical management: 2 cases of cardiac emerinopathy. JACC Clin Electrophysiol. 2021;7(3):410-412. doi:10.1016/j.jacep.2020.11.006 33516708

[11] Yoneda ZT, Anderson KC, Quintana JA, . Early-onset atrial fibrillation and the prevalence of rare variants in cardiomyopathy and arrhythmia genes. JAMA Cardiol. 2021;6(12):1371-1379. doi:10.1001/jamacardio.2021.3370 34495297 PMC8427496

[12] Fujita T, Fujino N, Anan R, . Sarcomere gene mutations are associated with increased cardiovascular events in left ventricular hypertrophy: results from multicenter registration in Japan. JACC Heart Fail. 2013;1(6):459-466. doi:10.1016/j.jchf.2013.08.007 24621997

[13] Olivotto I, Girolami F, Ackerman MJ, . Myofilament protein gene mutation screening and outcome of patients with hypertrophic cardiomyopathy. Mayo Clin Proc. 2008;83(6):630-638. doi:10.1016/S0025-6196(11)60890-2 18533079

[14] Pasotti M, Klersy C, Pilotto A, . Long-term outcome and risk stratification in dilated cardiolaminopathies. J Am Coll Cardiol. 2008;52(15):1250-1260. doi:10.1016/j.jacc.2008.06.044 18926329

[15] Ho CY. Genetics and clinical destiny: improving care in hypertrophic cardiomyopathy. Circulation. 2010;122(23):2430-2440. doi:10.1161/CIRCULATIONAHA.110.978924 21135371 PMC3100192

[16] Meregalli PG, Tan HL, Probst V, . Type of SCN5A mutation determines clinical severity and degree of conduction slowing in loss-of-function sodium channelopathies. Heart Rhythm. 2009;6(3):341-348. doi:10.1016/j.hrthm.2008.11.009 19251209

[17] Sacher F, Probst V, Iesaka Y, . Outcome after implantation of a cardioverter-defibrillator in patients with Brugada syndrome: a multicenter study. Circulation. 2006;114(22):2317-2324. doi:10.1161/CIRCULATIONAHA.106.628537 17116772

[18] Priori SG, Schwartz PJ, Napolitano C, . Risk stratification in the long-QT syndrome. N Engl J Med. 2003;348(19):1866-1874. doi:10.1056/NEJMoa022147 12736279

[19] McNally EM, Golbus JR, Puckelwartz MJ. Genetic mutations and mechanisms in dilated cardiomyopathy. J Clin Invest. 2013;123(1):19-26. doi:10.1172/JCI62862 23281406 PMC3533274

[20] Franklin by Genoox. Homepage. Accessed February 6, 2021. https://franklin.genoox.com/clinical-db/home

[21] Richards S, Aziz N, Bale S, ; ACMG Laboratory Quality Assurance Committee. Standards and guidelines for the interpretation of sequence variants: a joint consensus recommendation of the American College of Medical Genetics and Genomics and the Association for Molecular Pathology. Genet Med. 2015;17(5):405-424. doi:10.1038/gim.2015.30 25741868 PMC4544753

[22] Akinrinade O, Ollila L, Vattulainen S, . Genetics and genotype-phenotype correlations in Finnish patients with dilated cardiomyopathy. Eur Heart J. 2015;36(34):2327-2337. doi:10.1093/eurheartj/ehv253 26084686 PMC4561350

[23] Herman DS, Lam L, Taylor MR, . Truncations of titin causing dilated cardiomyopathy. N Engl J Med. 2012;366(7):619-628. doi:10.1056/NEJMoa1110186 22335739 PMC3660031

[24] Haas J, Frese KS, Peil B, . Atlas of the clinical genetics of human dilated cardiomyopathy. Eur Heart J. 2015;36(18):1123-35a. doi:10.1093/eurheartj/ehu301 25163546

[25] Maron BJ, Maron MS, Semsarian C. Genetics of hypertrophic cardiomyopathy after 20 years: clinical perspectives. J Am Coll Cardiol. 2012;60(8):705-715. doi:10.1016/j.jacc.2012.02.068 22796258

[26] Lee SP, Ashley EA, Homburger J, ; SHaRe Investigators. Incident atrial fibrillation is associated with MYH7 sarcomeric gene variation in hypertrophic cardiomyopathy. Circ Heart Fail. 2018;11(9):e005191. doi:10.1161/CIRCHEARTFAILURE.118.005191 30354366

[27] Butters A, Isbister JC, Medi C, . Epidemiology and clinical characteristics of atrial fibrillation in patients with inherited heart diseases. J Cardiovasc Electrophysiol. 2020;31(2):465-473. doi:10.1111/jce.14346 31930598

[28] Fatkin D, MacRae C, Sasaki T, . Missense mutations in the rod domain of the lamin A/C gene as causes of dilated cardiomyopathy and conduction-system disease. N Engl J Med. 1999;341(23):1715-1724. doi:10.1056/NEJM199912023412302 10580070

[29] Arbustini E, Pilotto A, Repetto A, . Autosomal dominant dilated cardiomyopathy with atrioventricular block: a lamin A/C defect-related disease. J Am Coll Cardiol. 2002;39(6):981-990. doi:10.1016/S0735-1097(02)01724-2 11897440

[30] van Tintelen JP, Hofstra RM, Katerberg H, ; Working Group on Inherited Cardiac Disorders, line 27/50, Interuniversity Cardiology Institute of The Netherlands. High yield of LMNA mutations in patients with dilated cardiomyopathy and/or conduction disease referred to cardiogenetics outpatient clinics. Am Heart J. 2007;154(6):1130-1139. doi:10.1016/j.ahj.2007.07.038 18035086

[31] Worman HJ, Bonne G. “Laminopathies”: a wide spectrum of human diseases. Exp Cell Res. 2007;313(10):2121-2133. doi:10.1016/j.yexcr.2007.03.028 17467691 PMC2964355

[32] Epp TA, Dixon IM, Wang HY, Sole MJ, Liew CC. Structural organization of the human cardiac alpha-myosin heavy chain gene (MYH6). Genomics. 1993;18(3):505-509. doi:10.1016/S0888-7543(11)80006-6 8307559

[33] Franco D, Lamers WH, Moorman AF. Patterns of expression in the developing myocardium: towards a morphologically integrated transcriptional model. Cardiovasc Res. 1998;38(1):25-53. doi:10.1016/S0008-6363(97)00321-0 9683906

