# Supplementary material for: Mortality Among Patients With Early-Onset Atrial Fibrillation and Rare Variants in Cardiomyopathy and Arrhythmia Genes
Source: JAMA Cardiol. 2022 May 11;7(7):733–41. doi: 10.1001/jamacardio.2022.0810 (PMC9096694; doi:10.1001/jamacardio.2022.0810)
Supplement: Supplement. — eAppendix 1. Description of the Registries eAppendix 2. Definitions for Baseline Demographics and Clinical Characteristics eTable 1. Gene List Table eAppendix 3. Mortality and Cause of Death Assessment eTable 2. ICD-10 Codes Listed as Primary or Contributing Causes of Death From NDI Records eAppendix 4. Statistical Methods eTable 3. Genes for Which Disease-Associated Variants Were Detected Grouped According to Inherited CM and Arrhythmia Syndromes eFigure 1. Adjusted Survival Curve for the Sensitivity Analysis That Restricted Disease-Associated Variants to Only Strong/Definitive Evidence Genes eFigure 2. Proportion of Participants With a Disease-Associated Variant According to LVEF at Enrollment eFigure 3. Kaplan-Meier Curve for Survival According to Disease-Associated TTN Variants eFigure 4. Kaplan-Meier Curve for Survival According to Disease-Associated MYH7 Variants eFigure 5. Kaplan-Meier Curve for Survival According to Disease-Associated LMNA Variants eFigure 6. Kaplan-Meier Curve for Survival According to Disease-Associated MYH6 Variants eFigure 7. Kaplan-Meier Curve for Survival According to Disease-Associated KCNQ1 Variants [file jamacardiol-e220810-s001.pdf]

Supplemental Online Content

Yoneda ZT, Anderson KC, Ye F, et al. Mortality among patients with early-onset atrial fibrillation and rare variants in cardiomyopathy and arrhythmia genes. *JAMA Cardiol.* Published online May 11, 2022. doi:10.1001/jamacardio.2022.0810

eAppendix 1. Description of the Registries

eAppendix 2. Definitions for Baseline Demographics and Clinical Characteristics

eTable 1. Gene List Table

eAppendix 3. Mortality and Cause of Death Assessment

eTable 2. ICD-10 Codes Listed as Primary or Contributing Causes of Death From NDI Records

eAppendix 4. Statistical Methods

eTable 3. Genes for Which Disease-Associated Variants Were Detected Grouped According to Inherited CM and Arrhythmia Syndromes

eFigure 1. Adjusted Survival Curve for the Sensitivity Analysis That Restricted Disease-Associated Variants to Only Strong/Definitive Evidence Genes

eFigure 2. Proportion of Participants With a Disease-Associated Variant According to LVEF at Enrollment

eFigure 3. Kaplan-Meier Curve for Survival According to Disease-Associated *TTN* Variants

eFigure 4. Kaplan-Meier Curve for Survival According to Disease-Associated *MYH7* Variants

eFigure 5. Kaplan-Meier Curve for Survival According to Disease-Associated *LMNA* Variants

eFigure 6. Kaplan-Meier Curve for Survival According to Disease-Associated *MYH6* Variants

eFigure 7. Kaplan-Meier Curve for Survival According to Disease-Associated *KCNQ1* Variants

This supplemental material has been provided by the authors to give readers additional information about their work.

© 2022 Yoneda ZT et al. *JAMA Cardiology.*

eAPPENDIX 1: Description of the registries

Vanderbilt Atrial Fibrillation Registry: The Vanderbilt Atrial Fibrillation (AF) Registry was started in 1999. It is approved by the Vanderbilt University Medical Center Institutional Review Board (IRB # 020669; PI: Roden). All participants undergo written, informed consent. Patients with AF and their family members are prospectively enrolled. At enrollment, a detailed past medical history is obtained along with an AF symptom severity assessment. Blood samples are obtained for DNA extraction. Patients are followed longitudinally to track clinic outcomes.

The Vanderbilt Atrial Fibrillation Ablation Registry (VAFAR) was started in 2011. It is approved by the Vanderbilt University Medical Center Institutional Review Board (IRB #110881; PI: Shoemaker). All participants undergo written, informed consent. It is a prospective observational registry of subjects undergoing AF ablation (clinicaltrials.gov NCT #02404415). Whole blood is collected during the ablation procedure from which DNA is extracted and stored along with serum and plasma. Baseline clinical data are manually extracted from the medical record and supplemented by patient interview. Pre-ablation imaging studies are performed (cardiac MRI or CT) and stored. Electrophysiologic data obtained at the time of ablation is collected and stored (data from the baseline electrophysiologic study and data and images from the electroanatomic map). Participants are prospectively followed for arrhythmia recurrence post-ablation and longitudinally to track longer term clinical outcomes.

© 2022 Yoneda ZT et al. *JAMA Cardiology.*

eAPPENDIX 2: Definitions for baseline demographics and clinical characteristics

Race and ethnicity were collected according to participant self-reporting. Hypertension was defined as prior diagnosis of hypertension, treatment with an antihypertensive medication not used for another indication, or 3 or more entries with systolic blood pressure >140 mmHg or diastolic blood pressure >90 mmHg. Valve disease was defined as a history of severe aortic, mitral, tricuspid or pulmonic insufficiency, or severe aortic stenosis, or mild or greater mitral stenosis. Myocardial infarction (MI) was defined as a history of an ST-elevation MI, a non-ST-elevation MI with revascularization, or myocardial scar identified by cardiac imaging.

© 2022 Yoneda ZT et al. *JAMA Cardiology.*

eTABLE 1: Gene list table (N=145 genes)

ABCC9	*DOLK*	*KCNE2*	*MTTS1*	*SCN1B*
ACTC1	*DSC2*	*KCNE3*	*MTTS2*	*SCN2B*
ACTN2	*DSG2*	*KCNE5*	*MYBPC3*	*SCN3B*
AGL	*DSP*	*KCNH2*	*MYH6*	*SCN4B*
AKAP9	*DTNA*	*KCNJ2*	*MYH7*	*SCN5A*
ALMS1	*EMD*	*KCNJ5*	*MYL2*	*SGCD*
ALPK3	*EYA4*	*KCNJ8*	*MYL3*	*SHOC2*
ANK2	*FHL1*	*KCNQ1*	*MYL4*	*SLC22A5*
ANKRD1	*FKRP*	*KRAS*	*MYLK2*	*SNTA1*
BAG3	*FKTN*	*LAMA4*	*MYOZ2*	*SOS1*
BRAF	*FLNC*	*LAMP2*	*MYPN*	*SOS2*
CACNA1C	*GAA*	*LDB3*	*NEBL*	*TAZ*
CACNA2D1	*GATA4*	*LMNA*	*NEXN*	*TBX20*
CACNB2	*GATA5*	*LRRC10*	*NKX2-5*	*TCAP*
CALM1	*GATA6*	*MAP2K1*	*NRAS*	*TECRL*
CALM2	*GATAD1*	*MAP2K2*	*PDLIM3*	*TGFB3*
CALM3	*GJA5*	*MIB1*	*PKP2*	*TMEM43*
CASQ2	*GLA*	*MTND1*	*PLEKHM2*	*TMPO*
CAV3	*GNB5*	*MTND5*	*PLN*	*TNNC1*
CAVIN4	*GPD1L*	*MTND6*	*PPA2*	*TNNI3*
CBL	*HCN4*	*MTTD*	*PRDM16*	*TNNT2*
CHRM2	*HFE*	*MTTG*	*PRKAG2*	*TOR1AIP1*
CPT2	*HRAS*	*MTTH*	*PTPN11*	*TPM1*
CRYAB	*ILK*	*MTTI*	*RAF1*	*TRDN*
CSRP3	*JPH2*	*MTTK*	*RANGRF*	*TRPM4*
CTF1	*JUP*	*MTTL1*	*RBM20*	*TTN*
CTNNA3	*KCNA5*	*MTTL2*	*RIT1*	*TTR*
DES	*KCND3*	*MTTM*	*RYR2*	*TXNRD2*
DMD	*KCNE1*	*MTTQ*	*SCN10A*	*VCL*

© 2022 Yoneda ZT et al. *JAMA Cardiology.*

eAPPENDIX 3: Mortality and cause of death assessment

For purpose of this analysis, vital status was censored up to 1/1/2020 and determined via two complementary methods. First, all participant records were reviewed up to the censor date for either: 1) mention of participant death prior to censoring or 2) follow up visit occurring after the censor date. Second, all participant records were sent to the NDI for record matching. The NDI is the most complete source of death information in the United States and holds all death records from 1979 for all 50 states and includes the corresponding ICD-10 codes for causes of death. Based on information provided, the NDI provides record matches for adjudicating matched death certificates to participants using a probabilistic match score (https://www.cdc.gov/nchs/data/ndi/NDI_Users_Guide.pdf).[1] Based on the matching algorithm, the NDI assigns each potential match a score. Using the NDI's cut-off score, the NDI records were used to confirm the vital status of all participants for whom the date of last follow-up occurred prior to the censor date or whose vital status remained unknown after chart review.

Cause of death information was obtained via the death certificate listed for all participants who were determined to be deceased. Death certificates list a "primary" and "contributing" cause (or causes) of death; however, the reporting differs among providers and among states. Therefore, deaths were considered as "CM-related," "sudden death," or "stroke-related," based on the inclusion of a corresponding ICD-10 code on either the primary or any of the contributing causes of death. A complete list of corresponding ICD-10 codes is presented in Supplemental Table 2.

REFERENCE:

1. Spector LG, Menk JS, Vinocur JM, et al. In-Hospital Vital Status and Heart Transplants After Intervention for Congenital Heart Disease in the Pediatric Cardiac Care Consortium: Completeness of Ascertainment Using the National Death Index and United Network for Organ Sharing Datasets. J Am Heart Assoc 2016;5(8). DOI: 10.1161/JAHA.116.003783.

© 2022 Yoneda ZT et al. *JAMA Cardiology.*

eTABLE 2: ICD-10 codes listed as primary or contributing causes of death from NDI records

Cardiomyopathy Death	Sudden Death	Stroke-related Death
I42.0 – Dilated cardiomyopathy<br>I42.1 – Obstructive hypertrophic cardiomyopathy<br>I42.2 – Other hypertrophic cardiomyopathy<br>I42.5 – Other restrictive cardiomyopathy<br>I42.6 – Alcoholic cardiomyopathy<br>I42.9 – Cardiomyopathy, unspecified<br>I50.0 – Congestive Heart Failure<br>I50.9 – Heart failure, unspecified<br>I51.6 – Cardiovascular disease, unspecified<br>I51.7 – Cardiomegaly<br>I51.8 – Other ill-defined heart diseases<br>I51.9 – Heart disease, unspecified	I45.8 – Other specified conduction disorders<br>I46.1 – Sudden cardiac death, so described<br>I46.9 – Cardiac arrest, unspecified<br>I47.2 – Ventricular tachycardia<br>I49 – Ventricular fibrillation and flutter<br>I49.5 – Sick Sinus Syndrome<br>I49.9 – Cardiac arrhythmia unspecified	I63.9 – Cerebral Infarction, unspecified<br>I64 – Stroke, not specified as hemorrhage or infarction<br>I67.9 – Cerebrovascular disease, unspecified<br>I69.8 – Sequelae of other and unspecified cerebrovascular diseases

© 2022 Yoneda ZT et al. *JAMA Cardiology.*

eAPPENDIX 4: Statistical methods

Results are reported at the participant level. Categorical variables are reported as frequency (N) and percentage, with continuous variables reported as the median and interquartile range (IQR). For between group univariate comparisons Mann-Whitney U tests (continuous variables) and Fishers' Exact tests (categorical variables) were used. The primary outcome for all analyses was death, measured in days, from time of enrollment to time of either death or censor date. The primary determinant was the presence or absence of a disease-associated variant. The primary analysis tested the hypothesis that a disease-associated variant increased the risk of death when adjusted for age at AF diagnosis, sex, race, BMI, LVEF, and the interaction between disease-associated variant status and age at AF diagnosis using a multivariable Cox regression model. If the interaction term was significant, the effect of a disease-associated variant alone was estimated using a likelihood ratio test that compared the full model with the interaction term to a reduced model without any terms that involved the disease-associated variant. The interaction between disease-associated variant status and age at AF diagnosis was graphically displayed (Figure 2 Panel B) where the "mortality risk" was calculated from the multivariable Cox model along with partial-effect plots generated from the model fitting results for visualization. Pre-specified secondary analyses grouped disease-associated variants according to 1) genes associated with specific inherited CM and arrhythmia syndromes, and 2) individual genes. A second pre-specified subgroup analysis tested the association between disease-associated variants and specific causes of death (CM-related and sudden death) using an adjusted multivariable Cox Proportional Hazards model. To avoid overfitting the multivariable model, there were fewer covariates used than in the primary analysis. The multivariable model for CM-related death adjusted for age at AF diagnosis, sex, race, and BMI. The multivariable model for sudden death adjusted for only age at AF diagnosis. Unadjusted Kaplan-Meier plots were presented along with log-rank hypothesis tests. Adjusted survival and cumulative incidence plots were presented, and P-values were calculated using the likelihood ratio test comparing the full model (adjusted for disease-associated variant, age, the interaction term of age and disease-associated variant, sex, race, BMI,

© 2022 Yoneda ZT et al. *JAMA Cardiology.*

and LVEF) to a reduced model that contained only age, sex, race, BMI, and LVEF. In the case of missing data, multiple imputation was used. Statistical analyses were performed using R version 4.0.0 (http://www.R-project.org) and Stata v.16 (StataCorp LLC, College Station, TX).

© 2022 Yoneda ZT et al. *JAMA Cardiology.*

eTABLE 3: Genes in which disease-associated variants were detected grouped according to related inherited CM and arrhythmia syndromes

ARVC/AC		Brugada Syndrome	CPVT	DCM			HCM		LQTS
DES	*MYL3*	*SCN5A**	*CASQ2**	*ACTC1*	*LMNA**	*SCN5A**	*ACTC1**	*MYL3**	*CACNA1C*
*DSP**	*SCN5A*			*CSRP3*	*MYBPC3*	*TPM1*	*CSRP3*	*TNNT2**	*KCNH2**
FLNC	*TTN*			*DES**	*MYH6*	*TTN**	*FHL1*	*TPM1**	*KCNQ1**
*JUP**				*DSP**	*MYH7**	*VCL*	*MYBPC3**	*TTN*	*SCN5A**
LMNA				*FHL1*	*NKX2-5*		*MYH6*	*VCL*	
MYH7				*FLNC**	*TNNT2**		*MYH7**		

*Genes that are designated by ClinGen (https://www.clinicalgenome.org) as having strong or definitive evidence. Not listed here are other genes not associated with traditional CM or arrhythmia syndromes, but a disease-associated variant was detected (*AKAP9, BRAF, CPT2, GATA4, GJA5, HRAS, KCNA5, MIB1, SCN3B, SOS2, TRPM4, TTR*).

© 2022 Yoneda ZT et al. *JAMA Cardiology*.

eFIGURE 1: Survival curve for the sensitivity analysis that restricted disease-associated variants to only strong/definitive evidence genes (N=104 participants). Adjustment was made for age at AF diagnosis, sex, race, BMI, LVEF, and an interaction term for disease-associated variant status and age ($P_{interaction}$=0.006). (P=0.005 likelihood ratio test).

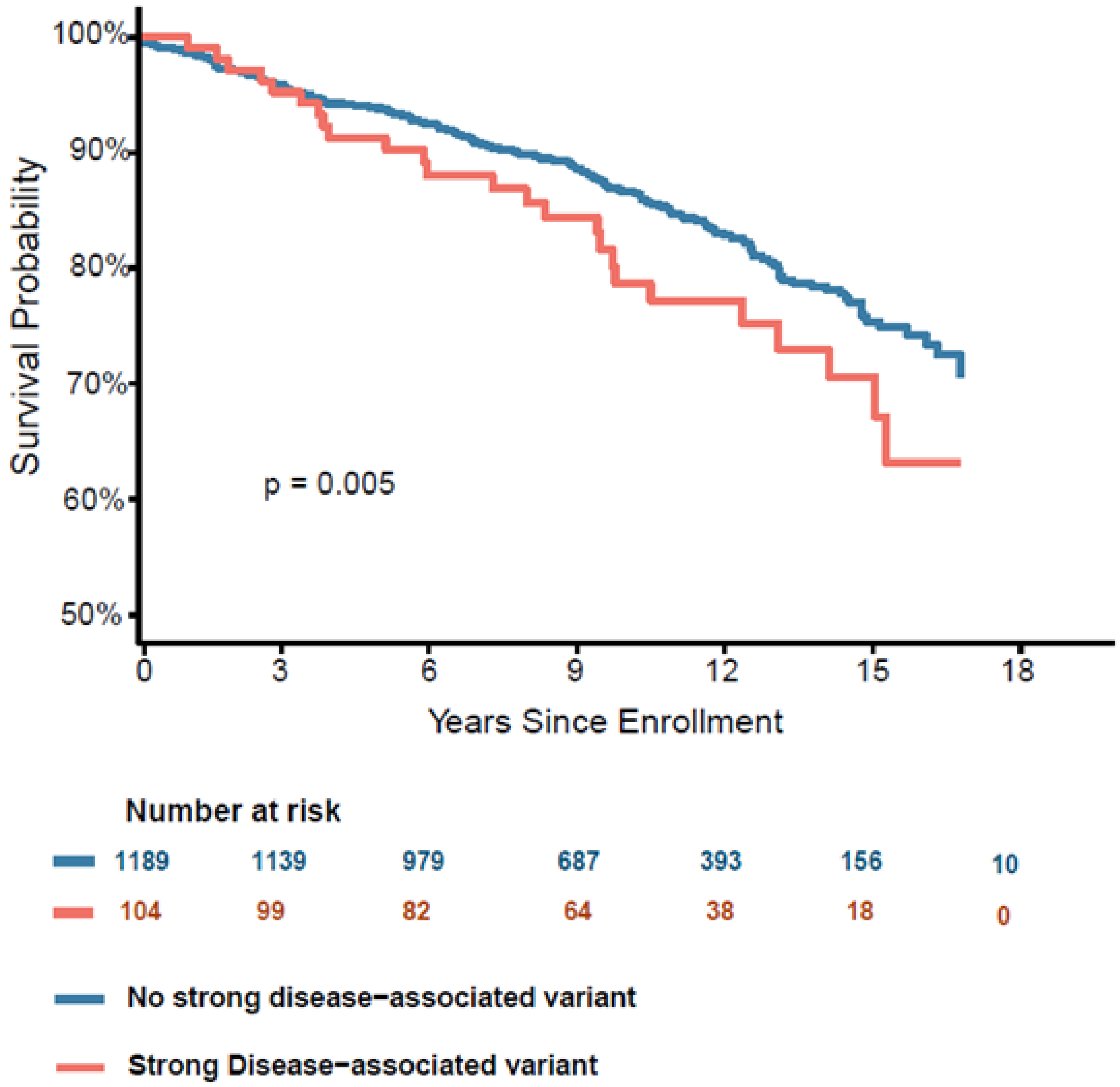

© 2022 Yoneda ZT et al. *JAMA Cardiology*.

eFIGURE 2: Proportion of participants with a disease-associated variant according to LVEF at enrollment. Most participants with depressed LV systolic function did not have a disease-associated variant. The number of participants with a disease-associated variant that died (all-cause mortality) during follow-up in the LVEF ≤39% group was 7, 40-49% group was 5, and ≥50% group was 18.

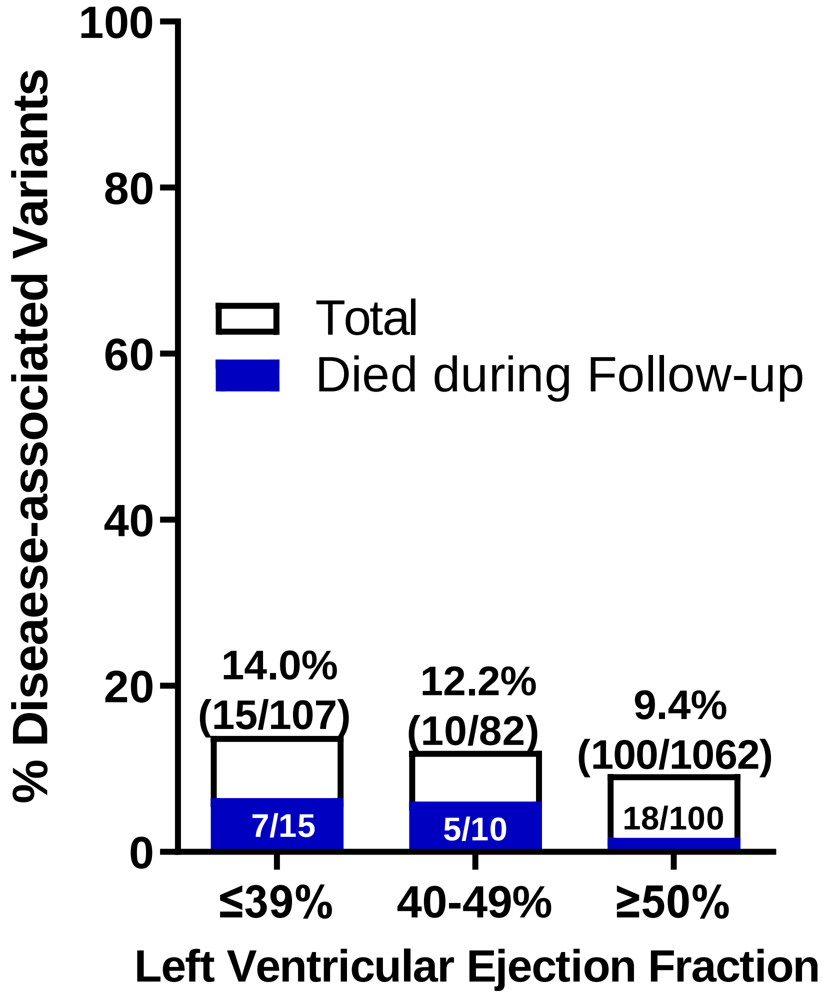

© 2022 Yoneda ZT et al. *JAMA Cardiology*.

eFIGURE 3: Kaplan-Meier curve for survival according to disease-associated *TTN* variants. P=0.31 by log-rank test.

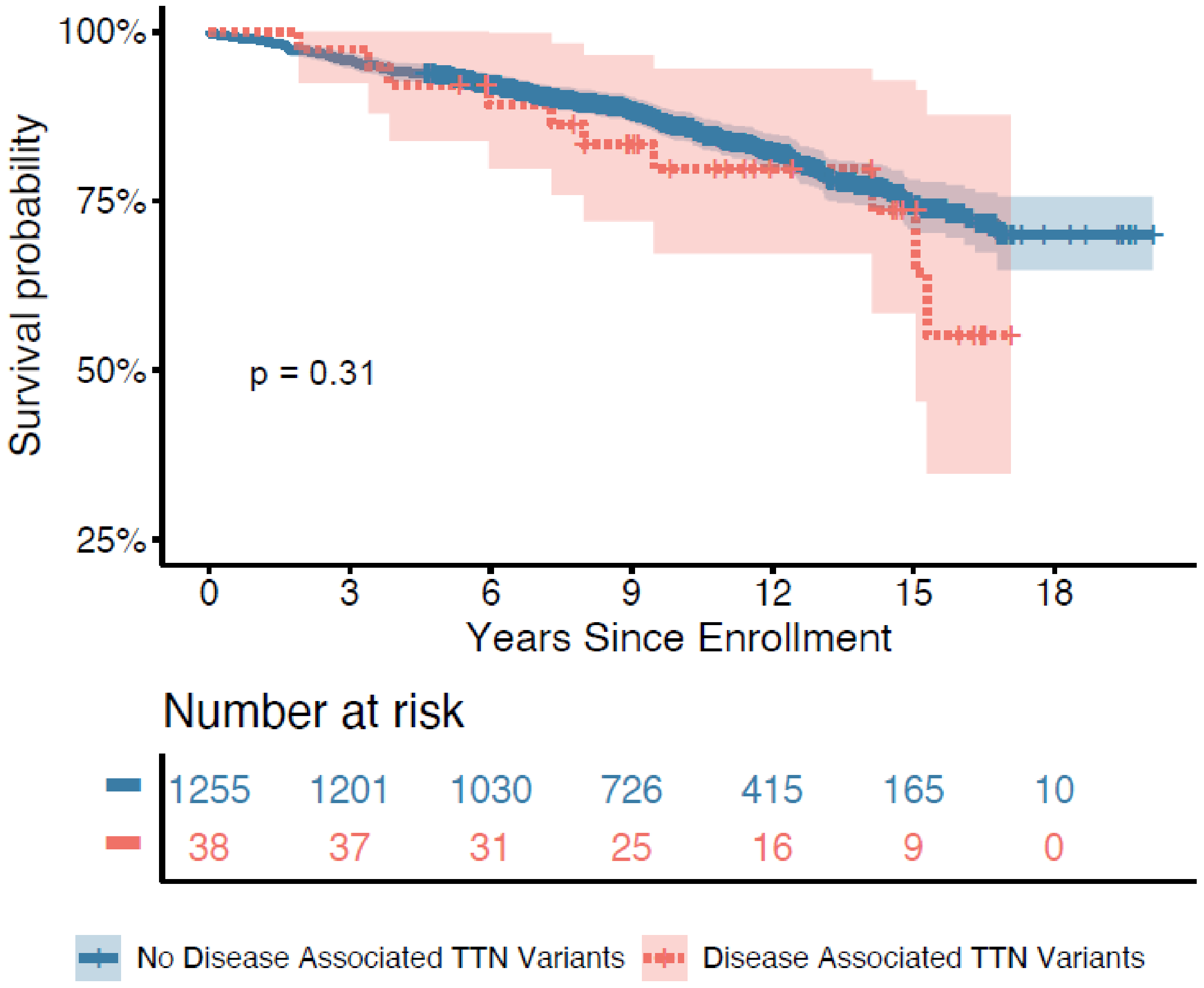

© 2022 Yoneda ZT et al. *JAMA Cardiology*.

eFIGURE 4: Kaplan-Meier curve for survival according to disease-associated *MYH7* variants. P=0.097 by log-rank test.

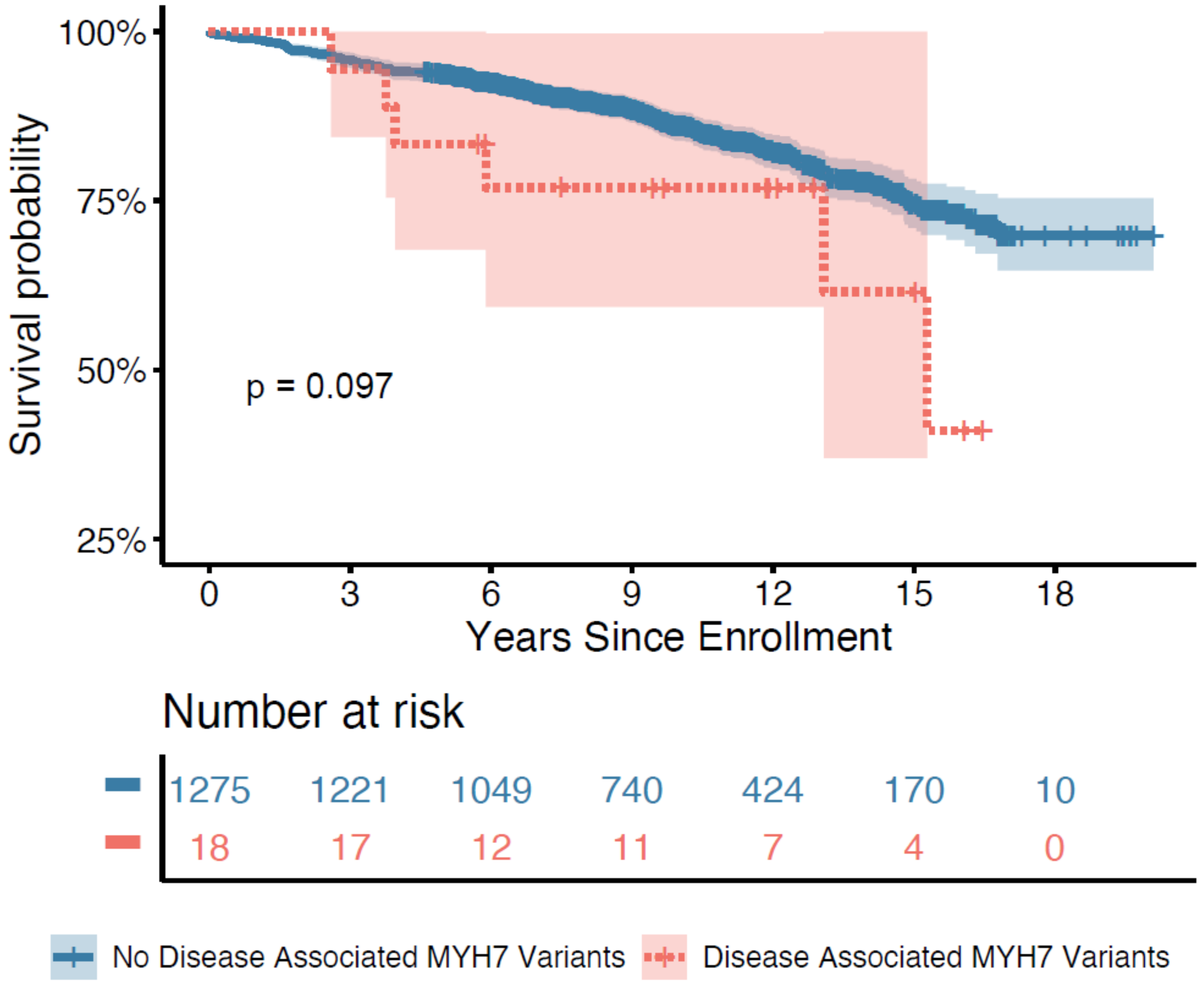

© 2022 Yoneda ZT et al. *JAMA Cardiology*.

eFIGURE 5: Kaplan-Meier curve for survival according to disease-associated *LMNA* variants. P=0.95 by log-rank test.

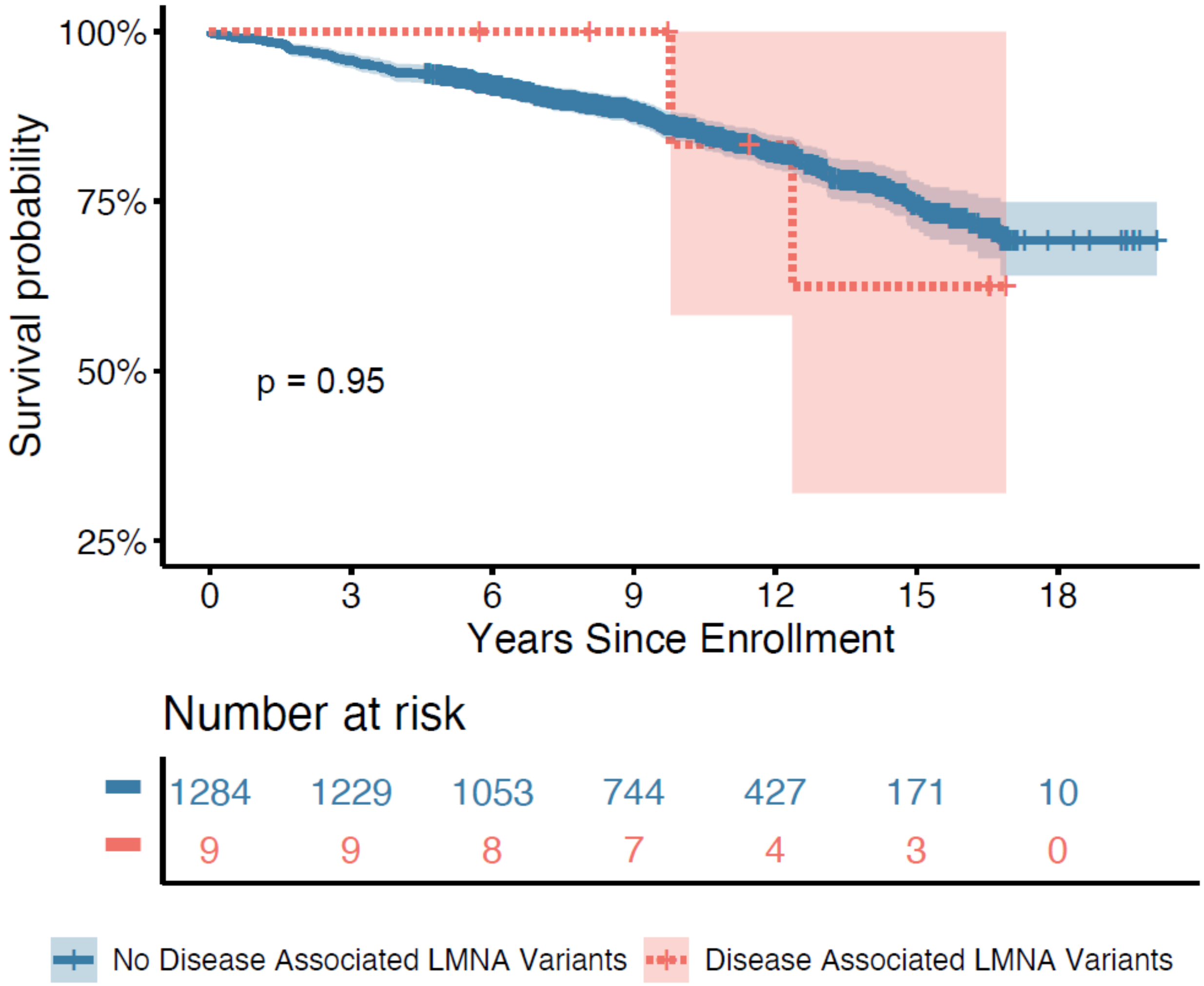

© 2022 Yoneda ZT et al. *JAMA Cardiology*.

eFIGURE 6: Kaplan-Meier curve for survival according to disease-associated *MYH6* variants. P=0.21 by log-rank test.

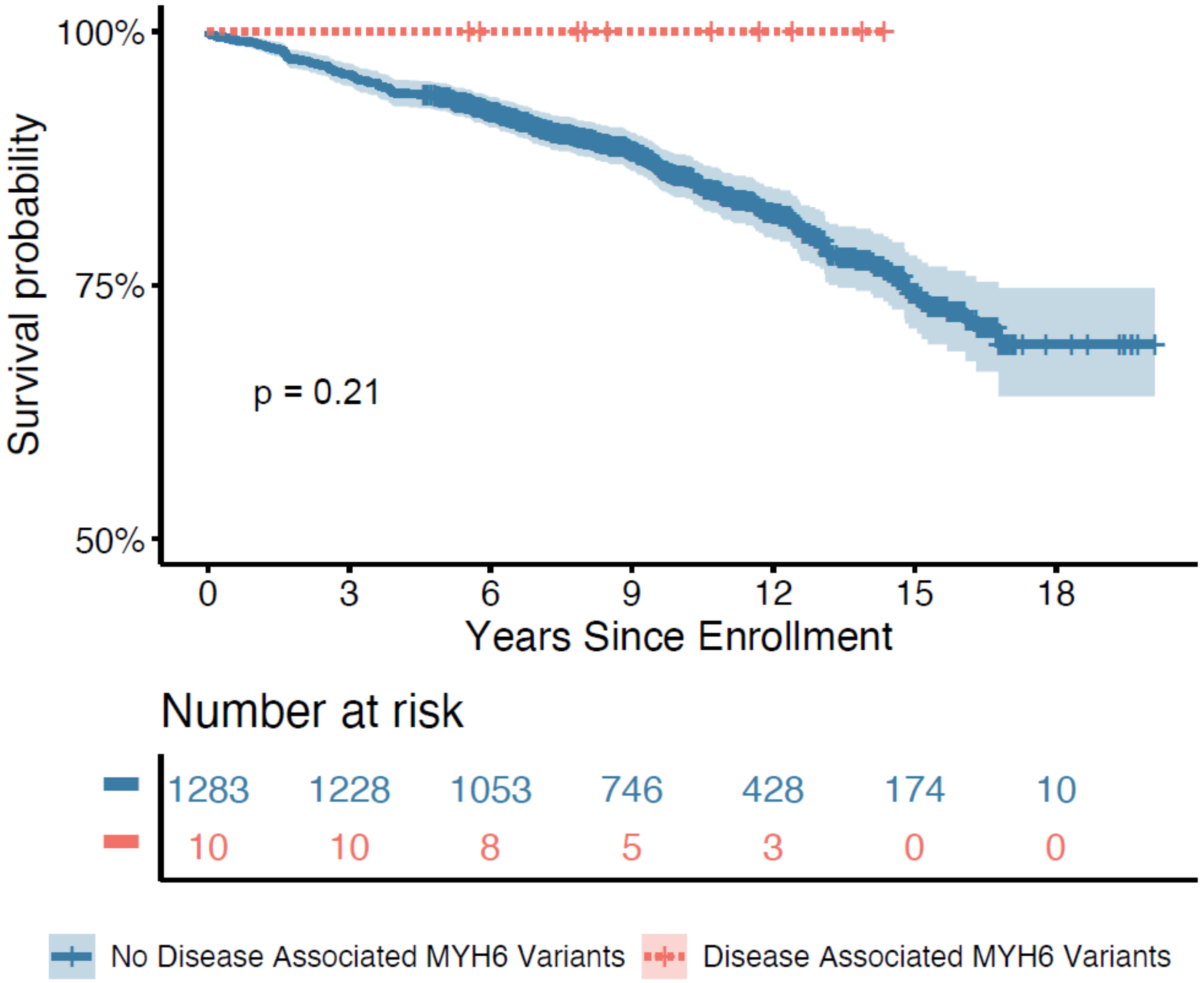

© 2022 Yoneda ZT et al. *JAMA Cardiology*.

eFIGURE 7: Kaplan-Meier curve for survival according to disease-associated *KCNQ1* variants. P=0.27 by log-rank test.

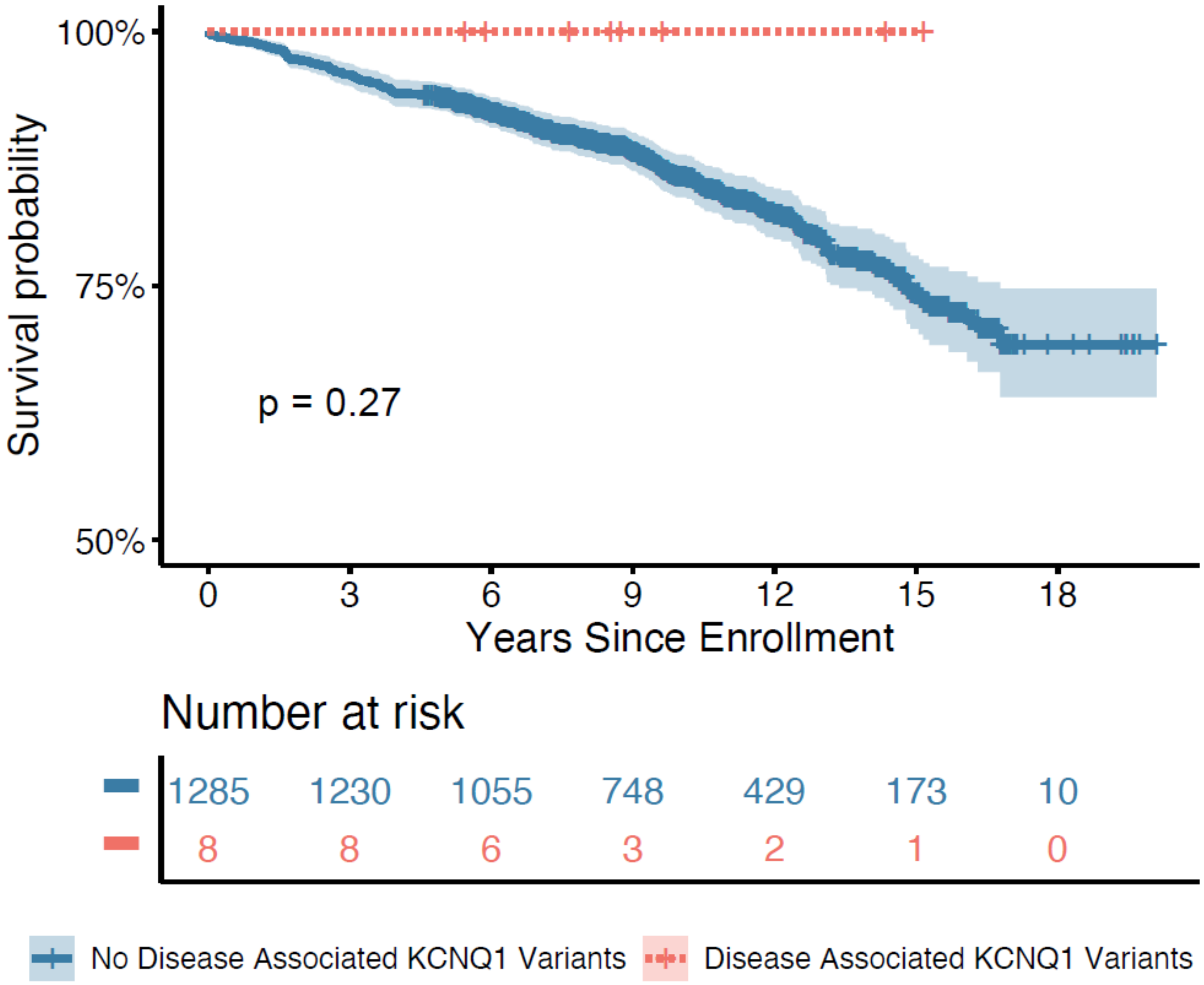

© 2022 Yoneda ZT et al. *JAMA Cardiology*.
